# An interview-based qualitative study of scalp cooling, alopecia, and disparities in Black breast cancer patients

**DOI:** 10.1007/s00520-026-10721-y

**Published:** 2026-05-05

**Authors:** Abena Minta, Lucy Rose, Olivia Ueltschi, Aliza Khuhro, Marisa Casola, Sonja Kobayashi, Sagar Sardesai, Brittany Dulmage

**Affiliations:** 1 The Ohio State University College of Medicine, Columbus, OH USA; 2 Department of Internal Medicine, Division of Medical Oncology, The Ohio State University Wexner Medical Center, Columbus, OH USA; 3 Department of Dermatology, The Ohio State University Wexner Medical Center, Columbus, OH USA

**Keywords:** Alopecia, Chemotherapy, Chemotherapy-induced alopecia, Scalp cooling, Supportive dermatologic care, Racial and ethnic disparities

## Abstract

**Background:**

Existing quality-of-life assessment tools for chemotherapy-induced alopecia may not adequately serve women of all races. Non-White patients with breast cancer receiving chemotherapy at our institution were approximately six times less likely than White patients to use scalp cooling (SC).

**Objective:**

This study examines factors contributing to this disparity through interviews with Black women undergoing chemotherapy, focusing on alopecia’s impact and attitudes toward its prevention and treatment.

**Methods:**

Semi-structured, 1-hour Zoom interviews were conducted and transcribed. Content analysis using NVIVO software and a grounded theory approach identified themes.

**Results:**

Three main domains emerged: (1) alopecia’s impact, (2) barriers to SC, and (3) improving alopecia management. Key barriers included limited representation of Black women in SC advertising and concerns about SC’s effectiveness on textured hair. Solutions included better counseling on SC use, camouflage options, and increased awareness of other treatment options like dermatology referrals.

**Limitations:**

The study was conducted at a single institution; participation was voluntary leading to possible selection bias and the risk for recall bias in the setting of assessing patients’ attitudes retrospectively.

**Conclusion:**

This study highlights barriers to SC use among Black women, providing insights for developing interventions to improve access to alopecia prevention and treatment.

**Supplementary Information:**

The online version contains supplementary material available at 10.1007/s00520-026-10721-y.

## Introduction

Research on breast health equity has focused on disparities in treatment and outcomes for minority women, with limited exploration of race-stratified adverse treatment effects [1–3]. Hair loss has gained attention in the survivorship literature, and quality-of-life assessment tools like the Chemotherapy-induced Alopecia Distress Scale (CADS) are emerging to measure its impact [4]. Additionally, researchers in the Netherlands have explored the utility of drawings to depict the cognitive and emotional impact of hair loss on patients in a mixed-methods qualitative cross-sectional study [5]. The design and validation of these instruments in populations with nontextured hair types raises reasonable concern regarding their ability to capture the Black patient experience [4, 5].

Similarly, although scalp cooling (SC) is the leading approach for prevention of chemotherapy-induced alopecia (CIA), cohort diversity in clinical trials has been limited; thus, providers and patients are uncertain about the benefit of scalp cooling in Black women who have a distinct hair texture and styling methods that can potentially impact scalp cooling efficacy [6–10]. The design of this qualitative, semistructured interview study was motivated by the results of a retrospective chart review at our institution exploring predictors of scalp cooling utilization in women with breast cancer that suggested non-White patients were approximately six times less likely to utilize scalp cooling compared to White patients (OR 0.15, 95% CI = 0.03 to 0.63, *p* = 0.01) [11]. To our knowledge, this is the first interpretive phenomenological study that aims to better understand the extent and impact of hair loss and to ascertain patient attitudes toward prevention and treatment of CIA in Black patients with breast cancer.

Given that Black patients are more likely to be treated with systemic chemotherapy due to advanced disease or aggressive biology, the burden of near-universal adverse hair loss is of interest in this patient population [12, 13].

## Methods

A retrospective sample of women with breast cancer who completed chemotherapy at the Ohio State University Comprehensive Cancer Center (OSUCCC) from 2018 to 2022 was identified. Key inclusion criteria included women aged 18 or older, identifying as Black or multiracial with Black ancestry, diagnosed with stages I–IV breast cancer, and self-reported new-onset hair loss while receiving chemotherapy. Exclusion criteria included a diagnosis of inflammatory hair loss. This study was approved by the institutional IRB (2023C0028).

Eligible participants were contacted for study enrollment via phone, offering a $50 gift card incentive. Informed consent was obtained through Research Electronic Data Capture (REDCap).

One-hour semistructured interviews were conducted via Zoom by a single interviewer (AM), using a mix of open- and closed-ended questions to allow for broad discussion of hair loss experiences and scalp cooling. To improve validity and reduce bias, a standard interview script developed with input from dermatologists, oncologists, and reviewed by the Tigerlily Foundation, a national women’s health and oncology organization providing support to young women with cancer. Targeted probing questions covered topics such as appearance concerns, quality of life impacts, alopecia treatments, and attitudes toward scalp cooling (full script found in Appendix 1).

Interviews were audio-recorded, transcribed, and analyzed using NVIVO software to identify themes and subthemes through grounded theory (GT) qualitative methodology with two-member authentication (BD and OU). GT is a research framework that utilizes theoretical saturation in three classic stages: (1) open coding (deriving concepts via a line-by-line data analysis); (2) axial coding (schematizing categories); and (3) selective coding (deriving core categories, describing a narrative outline) [14, 15].

## Results

To better understand the extent and impact of hair loss and to ascertain patient attitudes toward prevention and treatment of CIA, 17 Black women (mean age 50.16 years) meeting inclusion criteria were interviewed. Patient demographics are included in Table 1. Qualitative analysis of interview transcripts identified three major domains and several associated themes of the patient experience.

**Table 1:** Patient demographics

*Patient demographics (n* = *17)*	(mean) [range]
Age (years)	50.16 [35–69]
# of chemotherapy cycles	5.64 [1–9]
Breast cancer stage	***N* = **
Stage IA	4
Stage IIA	2
Stage IIIA	2
Stage IB	4
Stage IIB	4
Stage IV	1
Chemotherapy agent^*^	***N* = **
Taxane	11
Anthracycline	5
Herceptin	1

### Domain 1: effect of hair loss (Table 2)

**Table 2:** Effect of chemotherapy-induced hair loss on Black women

Domain 1: effect of hair loss	
*Themes:*	*Patient quotes:*
The effect of hair loss on family:	• “My children did not understand something was wrong until my hair fell out.” • “I had to cover my head whenever my kids were around. I didn’t want to be looking like I was sick in front of my kids.” • “When my son sees that the scarf is coming off, he’d [look up, point] and say ‘mom, mom check check’—like he doesn’t want it to come off to see my bald head.”
The effect of hair loss on mental health:	• ““[During chemotherapy], I tried to stay away from braids because I felt like they showed even more how many patches were on my scalp, but even after the chemo when my hair grew back I still changed the way that I styled it forever.” • “As women in particular, our hair is a lot of our self-image and how we perceive ourselves and how we want to be perceived… It’s kind of like a security blanket.” • “There is already the stigma that Black women ‘can’t grow hair’ or their hair isn’t as long as other races. So, when a black woman loses her hair, it’s devastating—her identity is tied to it.” • “As a female, [hair] is a part of our beauty. We take pride in our hair, the cancer process didn't bother me, but losing my hair was one of the side effects that really concerned me.”
The effect of hair loss on lifestyle:	• “One of the things that you don't want to do is be labeled as a cancer patient. Especially, for me at my age range as a corporate, professional and executive. So, I really had a concern about losing my hair—the change in my appearance—how people would treat me.” • “Working in it in a corporate setting, you're expected to present yourself a certain way and I bought into that. In the office, around the office in meetings—worse yet on Zoom meetings, I'm staring at myself the whole time and [hair loss] affected how I saw myself in the work setting.” • “Leaving the house looked like making sure my hair was in perfect shape, just to feel like everyone could see it on me—everyone could tell that my hair was thinner, or at least not like it was before. I didn’t want to go out.”

Overall, some patients reported good counseling on what to expect with hair loss and others did not, which may be attributed to differences in counseling styles or length of time spent on counseling. Specifically, unexpected negative emotions that patients reported were related to the rapid and diffuse nature of alopecia onset with one patient stating that, “I figured you shed hair a little bit at a time—maybe a month or so. I was prepared to just gradually go with it, but it was coming out in handfuls.” For patients with patchy alopecia or hair thinning, concern regarding the incompatibility of preferred protective styling and chemotherapy was common with one patient stating “[During chemotherapy], I tried to stay away from braids because I felt like they showed even more how many patches were on my scalp, but even after the chemo, when my hair grew back, I still changed the way that I styled it forever.” Further, some patients reported experiencing painful matting and tangling as hair was shed.

### Domain 2: barriers to scalp cooling among Black women (Table 3)

**Table 3:** Barriers to scalp cooling uptake among Black women

Domain 2: barriers to scalp cooling among Black women	
*Themes:*	*Patient quotes:*
Limited representation of Black women in advertising and online searches:	• “ I started looking at [scalp cooling], but I didn’t see a lot of African American women utilizing it. And if so they were more probably mixed race, which didn’t provide the total confirmation that [they] had a total understanding of true Black women with my hair.” • “When it comes to Black women and women of color, there’s just not a lot of representation so if we do have treatment options, there is always the concern that it is not ‘one size fits all’ and we usually find ourselves without any options.” • “In the brochure, there were very few women of color in there.” • “I think more African American women need to know about it. But I do think that the manual need to be a little more specific to all types of hair.”
Concern about efficacy in black women:	• “I didn’t see any Black people that succeeded in it.” • “I didn't know whether or not it would work on Black people.” • “I read about it, and Black hair didn’t have as good of a result. And first I'd even actually said yes, I’ll do it. After I read and researched and the thought of being cold on my head, I cancelled and said I didn’t want to do it.”
Concerns on possible adverse effects of cooling:	• “The red flag for me was the degree of coolness required, especially considering it’s your scalp and around your neck area. The chemo process itself already took some mental capacity, and I couldn’t see myself underneath the dome.” • “My perception of cold on my brain was it might make me not think as sharply, so that was a deterrent for me.”
Concerns about out-of-pocket cost:	• “I couldn’t afford to do scalp cooling. So I just lost all my hair.” • “My insurance ended up covering it—but if it didn’t, and for women who don’t have that—I think a definite barrier is that it’s pretty expensive.” • “The out of pocket cost for [SC] was crazy. My thought was [I] can buy about 15 wigs in comparison to trying to save hair that maybe couldn’t be saved—I wanted to understand what type of texture I was going to have or whether I was going to be bald [after scalp cooling], so the uncertainty, the ratio was the discouragement.”

An additional goal of the study was to determine barriers to scalp cooling uptake, and we identified four themes within this domain:

- *Theme 1:* limited representation of Black women in advertising and online searches
  Following scalp cooling discussions with their respective oncology teams, patients reported utilizing manufacturer advertising (e.g., manuals and websites) or online searches (e.g., Google and YouTube) to gather further information prior to deciding on scalp cooling use. The limited representation of Black women in these resources was a notable barrier, with one patient stating “I started looking at [scalp cooling], but I didn’t see a lot of African American women utilizing it. And if so, they were more probably mixed race, which didn’t provide the total confirmation that [they] had a total understanding of true Black women with my hair.”
- *Theme 2:* concern about efficacy in Black women
  The second theme we identified within this domain was concern regarding scalp cooling success in type IV/textured hair. Exemplifying this are patient quotes regarding “[not] seeing any Black people that succeeded in it” and “[not] knowing whether or not it would work on Black people.” Further, one patient stated “As Black women we tend to wear different styles and put different products in our hair. After reading and watching videos, I noticed [the instructions] encourage you to minimize that.” Significantly, one patient reported that after conducting an evidence-based search resulting in limited or unfavorable outcomes in patients with afro-textured hair, the adverse effects of cooling (our next identified theme) outweighed the probability of hair retention resulting in her returning the cold cap and opting out of scalp cooling. Approximately 57% of patients in our interview cohort who completed scalp cooling (4 out of 7) were reportedly satisfied with their results.
- *Theme 3:* concerns on possible adverse effects of cooling
- As patients discussed barriers to scalp cooling utilization, concern regarding coldness was common. Beyond the physical discomfort associated with the scalp temperature optimal for scalp cooling, patients expressed concern of iatrogenic cognitive affects.
- * Theme 4:* cost of scalp cooling
- Of the 17 women in our cohort, 10 women did not utilize scalp cooling (8 declined SC, 1 patient said yes then declined, 1 patient reported not being counseled). When discussing factors that contributed to this decision, cost was mentioned frequently. One patient stated “The out-of-pocket cost for [SC] was crazy. My thought was [I] can buy about 15 wigs in comparison to trying to save hair that maybe couldn’t be saved—I wanted to understand what type of texture I was going to have or whether I was going to be bald [after scalp cooling], so the uncertainty, the ratio was the discouragement.” Notably, for Black patients, perceived positive outcome may be influenced by retention of both hair density and hair texture.

### Domain 3: ways to improve CIA management (Table 4)

**Table 4:** Ways to improve CIA management in Black women

Domain 3: ways to improve CIA management	
*Themes:*	*Patient quotes:*
Theme 1:Tailored instructions on how to prepare the scalp and hair for cooling for those who elect to pursue it: Reported self-modifications for hair preparation:	• “I do think that the manual needs to be a little more specific to all types of hair” • “So, my understanding of Black hair, it needs to be flooded up with gel to go under the cool cap compared to White women.” • “They provided us some type of cream or a conditioner to put on your hair before you had your treatment, I didn’t use that—I just used a thicker leave in conditioner that I had in my house that I know would moisturize my hair.” • “I cut my hair, and I did not have the fro so the coolness could penetrate and improve the effectiveness.” • “It’s pretty labor intensive—especially if you have longer hair as the preparation requires parting your hair in many sections and [using a spray bottle] to wet it down, I had a second person help me, I don’t think it’s something you can easily do by yourself initially.”
Theme 2:Improved access to wigs that match their hair type:	• Regarding the wigs at CCC wig boutiques: “Their stuff just is not for Black people and I can’t, I can’t stress that enough.” • “I went to the wig boutique, and a lot of the wigs looked like White women’s hair. They don’t look like Black people’s hair. So I’d buy one and think ‘this looks fake,’ so I'd get another one…and another, to try to make it similar to what I lost.” • “The boutiques wigs or the wigs that they donated to me—none of them were suited for me, they weren’t geared toward people like me.”
Theme 3: Increased awareness of other treatment options:	• “I don’t know about any other treatment options. I mean, there’s scalp cooling or wigs, but other than that, I don’t even know what they are.” • “I think they should have referred me to the dermatologist. So they could of said well, you know, we got these options that you can get done that might help you not lose your hair. So, but without you knowing that, you just go through, lose your hair. It’s like you're going to lose your hair and that’s it.”
Theme 4: Improved insurance coverage or financial assistance for scalp cooling:	• “Because my insurance didn’t cover [scalp cooling] and it was a higher amount of money—I wasn’t willing to take the chance.” • “I just knew that it wasn’t covered by my insurance, so I didn’t look further into it.” • “I looked into a lot of resources, and you there’s not very many like programs or scholarships or anything like that, that can help with that price. If your insurance doesn’t cover it, then that’s what you pay.”

The final objective of this study was to identify ways to improve CIA management. Improved counseling on how to use scalp cooling with textured hair, access to camouflage options following hair loss, financial support for scalp cooling, and increased awareness of other treatment options including dermatology referral were identified as themes within this domain.

- *Theme 1:* tailored instructions on how to prepare the scalp and hair for cooling for those who elect to pursue it
  For Black women who elected to undergo scalp cooling, the addition of styling products and instructions tailored for textured/afro hair was commonly identified as a way to improve the patient experience. One patient stated, “I could tell that the [scalp cooling] process was not designed for African American hair because the manual had language such as ‘lather your long’ … that was straight-hair-framed, and there was no mention of curliness. There was an inherent assumption that your hair was thin and would be receptive to the conditioner that they provide or that the cooling cap will automatically fit to your hair. For a non-African American woman, with straight hair, if wet, it’s likely flat. Whereas for an African American woman, if you wet your hair, your hair grows up, and the cap does not necessarily sit on your head as secure.” Furthermore, patients frequently described self-modifications to improve afro penetration such as cutting their hair short into a “pixie” style, applying generous amounts of hair gel prior to scalp cooling, and using an alternative conditioner/leave-in as the consistency of the scalp cooling preparation products provided lacked moisture.
- *Theme 2:* improved access to racially inclusive wigs
  For patients seeking cost-effective alternatives to scalp cooling or who fail scalp cooling, alopecia camouflage with cranial prostheses may be considered. Patients in our cohort reported searching for cranial prostheses at the Comprehensive Cancer Center’s wig boutique, which may help with insurance coverage and has a small selection of pro-bono units for patients. However, the utility of this service to Black women may be limited as patients frequently described the absence of wigs similar in texture or style to their natural hair.
- *Theme 3:* improved insurance coverage or financial assistance for scalp cooling
  Overwhelmingly, improved insurance coverage and financial assistance for scalp cooling were identified by patients in our cohort as strategies to improve CIA counseling. Several women categorized both hair and breast loss as adverse effects of chemotherapy, suggesting that insurance coverage of the “reconstruction phase” should include hair retention, camouflage and/or regrowth. For many patients in our cohort, fears of scalp cooling failure were exacerbated by the associated out-of-pocket cost. As one patient stated, “Because my insurance didn’t cover [scalp cooling] and it was a higher amount of money—I wasn’t willing to take the chance.”
- *Theme 4:* increased awareness of other treatment options
  Excluding scalp cooling or alopecia camouflage, most patients in our cohort were unaware of additional management options for CIA. Some patients expressed feelings of helplessness; following an expression of the financial constraints of scalp cooling, there was limited discussion of other therapeutic options. For example, 15 of the 17 patients (88.2%) reported they were not familiar with minoxidil. Against this background, several patients suggested that early referral to dermatology for pharmacologic therapy should be considered. The importance of centralized interdisciplinary collaboration to manage the adverse effects of oncology therapy is captured by a patient who stated, “I think that it would be great to have a packet or even a team of experts for different areas [of concern], with a dermatologist inclusive on the team.”

## Discussion

Previous studies have found that the burden of physical symptoms associated with chemotherapy may hinder treatment adherence in some patients with breast cancer [12, 13]. Evaluating supportive oncology services for marginalized groups could help address disparities in chemotherapy discontinuation, as the literature suggests institutional services targeting appearance-related side effects can improve patients’ mood and quality of life [16, 17]. Thematic analyses of the data collected from 17 Black women with a history of breast cancer revealed key recommendations to improve the overall management of CIA (Fig. 1).

For both scalp-cooled and nonscalp-cooled women, the general lack of representation of Black women in scalp cooling–related advertising was a major barrier that reduced patients’ optimism regarding scalp cooling effectiveness in curly or coily textured hair types. Corroborating these concerns, a study evaluating representation in pharmaceutical drug advertising found that ads featuring only Black individuals were less likely to address serious health conditions compared to those featuring only White individuals [18]. Given the increased likelihood of Black women receiving chemotherapy and subsequent chemotherapy-induced alopecia, there is an ethical responsibility to ensure health-related advertising captures high-risk patients [16, 17].

Scalp cooling expenses range from $1347 to $2245, and while insurance coverage is improving, many patients still face out-of-pocket expenses [19, 20]. Nonprofit organizations provide application-based scholarships to help cover these costs, but patients often remain unaware of this assistance, highlighting the need for healthcare providers to inform them about available financial resources [21, 22]. In our study, while cost was a significant barrier for women who did not use scalp cooling, those who did expressed more concerns about its efficacy. Given the limited representation of Black women in scalp cooling resources, patients may place heightened significance in physician counseling [23, 24]. Further, previous studies have shown that physicians may make premature assumptions about non-White patients’ compliance and ability to pay, limiting the counseling and treatment options offered [6, 25]. Thus, physicians and other healthcare professionals should work to reduce unconscious bias, and future clinical trials should include diverse hair textures to enhance evidence-based counseling.

When counseling Black women at risk for CIA, physicians and other members of the care team (e.g., nurse navigators) should adopt culturally relevant approaches to Black hair [23]. Since Black women frequently alternate hairstyles, gathering information about the patient’s current hair practices, such as whether their hair is natural or chemically relaxed, may improve joint assessment of the scalp cooling feasibility. Preparation for scalp cooling differs. Women with relaxed hair can wet their hair per manufacturer instructions, while those with natural hair should avoid excess wetting, opting instead to straighten their hair and apply emollients [6, 9]. Consideration of time and fatigue constraints for straightening natural hair and the potential damage from frequent heat application is important. Additionally, chemical relaxers can weaken hair strands, increasing the risk of diffuse, permanent alopecia due to higher baseline hair fragility. Assessing how often patients use protective styles is also crucial, as changes in hair texture reported by some Black patients postchemotherapy may not tolerate the weight associated with these styles [6, 9, 23]. Furthermore, previous studies have demonstrated the positive impact of community-based initiatives that promote health education through partnerships with trusted nonmedical professionals in the Black community, such as cosmetologists and hair stylists [24, 25]. Given the influence of hair stylists in the Black community, oncology teams should consider collaborating with stylists to enhance education on patients’ hair health and styling practices.

For patients who choose not to use scalp cooling, oncology teams should be aware of alternative alopecia management options, such as hair camouflage and pharmacologic therapy. Cranial prostheses are an alternative with potential insurance coverage. However, our cohort noted a lack of racially inclusive wigs that resemble natural hair at the affiliated wig boutique, prompting a survey of Comprehensive Cancer Center wig boutiques across the United States. This survey revealed a nationwide shortage of kinky/textured wigs, disproportionately impacting Black patients, who may have to turn to noncovered online vendors [26]. Additionally, a systematic review indicated that patients often feel unprepared for alopecia due to limited information and resources to mitigate the psychological burden of hair loss [27]. To alleviate this, oncologists should consider timely referrals to dermatology for pharmacologic therapy.

## Conclusion

This qualitative study examined the impact of hair loss on Black women with breast cancer, focusing on their attitudes toward scalp cooling and counseling for CIA. Participants noted a lack of information on scalp cooling tailored to Black women, highlighting the need for customized guidance. Financial barriers were the primary concern for those who did not use scalp cooling, while efficacy and hair texture changes influenced the decisions of those who did. A notable barrier was the limited representation of Black women in scalp cooling marketing. To improve CIA management for this population, we recommend a “texture-tailored” counseling approach that considers patients’ baseline styling practices. Interdisciplinary collaboration among oncologists, dermatologists, wig boutiques, and hairdressers could provide an optimal, patient-centered strategy.

## Supplementary Information

Below is the link to the electronic supplementary material.

Supplementary Material 1 (DOCX 21.4 KB)

## Data Availability

The participants of this study did not give written consent for their data to be shared publicly, so due to the sensitive nature of the research supporting data is not available.

## Abbreviations

CIA: Chemotherapy-induced alopecia
GT: Grounded theory
SC: Scalp cooling

## References

[1] Drake B, James A, Miller H et al (2022) Strategies to achieve breast health equity in the St. Louis Region and beyond over 15+ years. Cancers (Basel) 14(10):2550. 10.3390/cancers14102550 35626157 10.3390/cancers14102550 PMC9140077

[2] Marlow EC, Wysocki KL, Wehling KA et al (2023) Breast health equity along the cancer care continuum: a collaborative grant initiative. Cancer 129(S19):3084–3086. 10.1002/cncr.34595 37691527 10.1002/cncr.34595

[3] Glaser KM, Dauphin C, Johnson D et al (2023) Advancing community-academic partnerships to achieve breast health equity: applying the community-based participatory model to build capacity for sustained impact. Cancer 129(S19):3162–3170. 10.1002/cncr.34976 37691523 10.1002/cncr.34976 PMC10513749

[4] Cho J, Choi EK, Kim IR et al (2014) Development and validation of Chemotherapy-induced Alopecia Distress Scale (CADS) for breast cancer patients. Ann Oncol 25(2):346–351. 10.1093/annonc/mdt476 24379161 10.1093/annonc/mdt476

[5] van Alphen K, Versluis A, Dercksen W et al (2020) Giving a face to chemotherapy-induced alopecia: a feasibility study on drawings by patients. Asia Pac J Oncol Nurs 7(2):218–224. 10.4103/apjon.apjon_8_20 32478141 10.4103/apjon.apjon_8_20 PMC7233558

[6] Robinson W, McLellan BN (2023) Utilization of scalp cooling to prevent chemotherapy-induced alopecia in woman of skin of color with type 3 hair. JAAD Case Rep 50:65–68. 10.1016/j.jdcr.2023.10.018 39044995 10.1016/j.jdcr.2023.10.018 PMC11263471

[7] Komen MM, Smorenburg CH, van den Hurk CJ, Nortier JW (2013) Factors influencing the effectiveness of scalp cooling in the prevention of chemotherapy-induced alopecia. Oncologist 18(7):885–91. 10.1634/theoncologist.2012-0332 23650021 10.1634/theoncologist.2012-0332 PMC3720643

[8] Hershman DL (2017) Scalp cooling to prevent chemotherapy-induced alopecia: the time has come. JAMA 317(6):587–588. 10.1001/jama.2016.21039 28196237 10.1001/jama.2016.21039

[9] Araoye EF, Stearns V, Aguh C (2020) Considerations for the use of scalp cooling devices in Black patients. J Clin Oncol 38(30):3575–3576. 10.1200/JCO.20.02130 32897824 10.1200/JCO.20.02130 PMC7571797

[10] Dilawari A, Gallagher C, Alintah P et al (2021) Does scalp cooling have the same efficacy in Black patients receiving chemotherapy for breast cancer? Oncologist 26(4):292-e548. 10.1002/onco.13690 33512741 10.1002/onco.13690 PMC8018328

[11] Rose L, Schnell PM, Radcliff L, Lustberg M, Dulmage B (2023) Retrospective cohort study of scalp cooling in breast cancer patients. Support Care Cancer 31(2):118. 10.1007/s00520-022-07562-w 36645520 10.1007/s00520-022-07562-w

[12] Stringer-Reasor E et al (2021) Disparities in breast cancer associated with African American identity. Am Soc Clin Oncol Educ Book 41:e29–e46. 10.1200/EDBK_319929 34161138 10.1200/EDBK_319929

[13] Chen JC et al (2024) Racial disparities in breast cancer: from detection to treatment. Curr Oncol Rep 26(1):10–20 38100011 10.1007/s11912-023-01472-8

[14] Im D, Pyo J, Lee H et al (2023) Qualitative research in healthcare: data analysis. J Prev Med Public Health 56(2):100–110 37055353 10.3961/jpmph.22.471 PMC10111102

[15] Strauss AL, Corbin JM (1990) Basics of qualitative research: grounded theory procedures and techniques. Sage, Newbury Park

[16] Xing CY, Doose M, Qin B, Lin Y, Carson TL, Plascak JJ, Demissie K, Hong CC, Bandera EV, Llanos AAM (2020) Pre-diagnostic allostatic load and health-related quality of life in a cohort of Black breast cancer survivors. Breast Cancer Res Treat 184(3):901–914. 10.1007/s10549-020-05901-1 32914357 10.1007/s10549-020-05901-1 PMC7657984

[17] Ferreira MN, Ramseier JY, Leventhal JS (2019) Dermatologic conditions in women receiving systemic cancer therapy. Int J Womens Dermatol 5:285–307 31909148 10.1016/j.ijwd.2019.10.003 PMC6938835

[18] Ball JG, Liang A, Lee WN (2009) Representation of African Americans in direct-to-consumer pharmaceutical commercials: a content analysis with implications for health disparities. Health Mark Q 26(4):372–390. 10.1080/07359680903304328 19916100 10.1080/07359680903304328

[19] Cold cap therapy: cost, insurance and financial assistance. ucsfhealth.org. Published April 22, 2024. https://www.ucsfhealth.org/education/cold-caps-cost-insurance-financial-assistance. Accessed 2 Oct 2024

[20] Insurance reimbursement for scalp cooling. Paxman. Published November 2, 2023. https://coldcap.com/help-me-decide/accessing-scalp-cooling/. Accessed 2 Oct 2024

[21] Apply for funding. Cap & conquer. Published 2021. https://capandconquer.org/apply-for-funding. Accessed 2 Oct 2024

[22] Paterson C, Kozlovskaia M, Turner M et al (2021) Identifying the supportive care needs of men and women affected by chemotherapy-induced alopecia? A systematic review. J Cancer Surviv 15(1):14–28. 10.1007/s11764-020-00907-6 32683651 10.1007/s11764-020-00907-6

[23] Pleasant VA et al (2023) Redefining the ‘Crown’: approaching chemotherapy-induced alopecia among Black patients with breast cancer. Cancer 129(11):1629–1633. 10.1002/cncr.34732 37158640 10.1002/cncr.34732

[24] Sadler GR, Ko CM, Wu P, Alisangco J, Castañeda SF, Kelly C (2011) A cluster randomized controlled trial to increase breast cancer screening among African American women: the Black cosmetologists promoting health program. J Natl Med Assoc 103(8):735–745. 10.1016/s0027-9684(15)30413-2 22046851 10.1016/s0027-9684(15)30413-2 PMC4153602

[25] Madigan ME, Smith-Wheelock L, Krein SL (2007) Healthy hair starts with a healthy body: hair stylists as lay health advisors to prevent chronic kidney disease. Prev Chronic Dis 4(3):A64 17572968 PMC1955408

[26] Minta A, Rose L, Shareef SJ, Adame S, Dulmage B (2024) Availability of cranial prostheses for Black patients at comprehensive cancer centers. JCO Oncol Pract 20(10):1420–1425. 10.1200/OP.23.00750 38917378 10.1200/OP.23.00750

[27] Peterson LL, Lustberg M, Tolaney SM et al (2020) Integration of physician and nursing professional efforts to deliver supportive scalp cooling care to oncology patients at risk for alopecia. Oncol Ther 8(2):325–332. 10.1007/s40487-020-00120-6 32700046 10.1007/s40487-020-00120-6 PMC7683634

