# Supplementary material for: An interview-based qualitative study of scalp cooling, alopecia, and disparities in Black breast cancer patients
Source: Support Care Cancer. 2026 May 5;34(6):498. doi: 10.1007/s00520-026-10721-y (PMC13144178; doi:10.1007/s00520-026-10721-y)
Supplement: Supplementary file 1 — Supplementary Material 1 (DOCX 21.4 KB) [file 520_2026_10721_MOESM1_ESM.docx]

APPENDIX 1: INTERVIEW SCRIPT

Introduction:

Thank you so much for volunteering your time today to participate in this interview. My name is [name], I am [role on research team]. The goal of this study is to identify the extent and impact of hair loss on Black women undergoing chemotherapy for breast cancer.

As an overview, this interview will have both open and closed-ended questions. I encourage you to elaborate as much as you feel comfortable and introduce new concepts if there is anything that you would like to expand on. As a reminder, we will be recording this interview and later transcribing the recording so we can make sure we are accurately capturing and amplifying your voice.

The interview will take no more than 1 hour and you will receive a $50 gift card for your time.

Do you have any questions before we begin?

Interview questions:

At what point did you notice your hair loss during treatment?

How has hair loss from chemotherapy impacted your life?

How did you feel about hair loss prior to starting chemotherapy?

How did you feel about hair loss during chemotherapy?

How did you feel about hair loss after chemotherapy?

Did hair loss from chemotherapy impact your social life?

Do you think hair loss has impacted how you were perceived or treated by others?

Did hair loss from chemotherapy impact your work?

Did you choose to wear a wig or other hair piece? What was that experience like?

How did you cope with hair loss?

Were there other coping mechanisms used regarding hair loss like specific products, or techniques?

What is the significance of hair loss to you?

Did you have a conversation with you oncologist about potential hair loss prior to treatment?

Tell me about your conversation with your oncologist about expected hair loss prior to treatment.

Did the likelihood of hair loss with chemotherapy affect your decision to pursue a specific treatment regimen?

Did you discuss hair loss management approaches with your oncology team?

Did you discuss the expected degree of hair loss with your oncology team?

Aside from your oncology team, were there other resources you used to learn about hair loss from chemotherapy?

Were you referred to a dermatologist, or another professional or resource, for hair loss evaluation or treatment at any point?

If yes, can you tell me about that experience?

Have you heard of scalp cooling before? What do you know about scalp cooling?

Did you discuss scalp cooling with your oncology team?

If yes, can you tell me about that conversation?

Did you know of other individuals or friends that completed scalp cooling? Did they consider it to be successful for them?

Did your oncologist describe how the effectiveness of scalp cooling would be with your hair type/texture?

Did you choose to undergo scalp cooling?

If yes, why did you choose to undergo scalp cooling?

If yes, what was your experience with scalp cooling?

If yes, how successful was scalp cooling for you?

If yes, did you have any side effects during or after scalp cooling?

If no, why did you decide against scalp cooling?

If no because scalp cooling was not introduced by oncology team, is scalp cooling an option you may have considered if it was offered?

If no related to inconvenience, what modifications to scalp cooling could make it a more feasible option?

Now I will ask you about some specific factors that might have influenced your decision to undergo scalp cooling.

Please indicate the level that you agree or disagree with the following statements. A rating of “1” means that you strongly agree, and the factor had a high influence on your decision to undergo scalp cooling. A rating of “5” means that you strongly disagree, and the factor had low influence on your decision to undergo scalp cooling.

1. The cost of scalp cooling influenced my decision to undergo scalp cooling

1-Strongly Agree 2-Agree 3-Neutral 4 -Disagree 5- Strongly Disagree

1. The time required for scalp cooling influenced my decision to undergo scalp cooling

1-Strongly Agree 2-Agree 3-Neutral 4 -Disagree 5- Strongly Disagree

1. My concerns about financial difficulties related to my cancer treatment influenced my decision to undergo scalp cooling

1-Strongly Agree 2-Agree 3-Neutral 4 -Disagree 5- Strongly Disagree

1. My concerns about my appearance and body image influenced my decision to undergo scalp cooling

1-Strongly Agree 2-Agree 3-Neutral 4 -Disagree 5- Strongly Disagree

1. My concerns about the impact of hair loss on my career influenced my decision to undergo scalp cooling

1-Strongly Agree 2-Agree 3-Neutral 4 -Disagree 5- Strongly Disagree

1. My concerns about the impact of hair loss on my social life influenced my decision to undergo scalp cooling

1-Strongly Agree 2-Agree 3-Neutral 4 -Disagree 5- Strongly Disagree

1. My concerns about the impact of hair loss on my anxiety influenced my decision to undergo scalp cooling

1-Strongly Agree 2-Agree 3-Neutral 4 -Disagree 5- Strongly Disagree

1. My concerns about the impact of hair loss on my depression influenced my decision to undergo scalp cooling

1-Strongly Agree 2-Agree 3-Neutral 4 -Disagree 5- Strongly Disagree

1. My concerns about scalp cooling success with my hair type/texture influenced my decision to undergo scalp cooling

1-Strongly Agree 2-Agree 3-Neutral 4 -Disagree 5- Strongly Disagree

1. My concerns about the possibility of failure of scalp cooling impacted my decision

1-Strongly Agree 2-Agree 3-Neutral 4 -Disagree 5- Strongly Disagree

1. I am satisfied with a wig or head covering for management of my hair loss after chemotherapy

1-Strongly Agree 2-Agree 3-Neutral 4 -Disagree 5- Strongly Disagree

1. The cost of a wig influenced my decision to undergo scalp cooling

1-Strongly Agree 2-Agree 3-Neutral 4 -Disagree 5- Strongly Disagree

1. I am satisfied with the information provided about scalp cooling by my oncology team

1-Strongly Agree 2-Agree 3-Neutral 4 -Disagree 5- Strongly Disagree

1. I am satisfied with the information provided about treatment options for my hair loss due to chemotherapy provided by my oncology team

1-Strongly Agree 2-Agree 3-Neutral 4 -Disagree 5- Strongly Disagree

Tell me about your feelings regarding treatments for hair loss.

Did you try anything to encourage or speed hair regrowth?

If yes, what did you try?

Have you heard of minoxidil or Rogaine?

If yes, how did you hear about it?

If yes, what did you know about it?

If yes, did you try using it?

If yes, what was your experience?

Do you think your hair has recovered from chemotherapy?

How do you feel that your hair has recovered from chemotherapy?

Do you wear a wig or other hair covering more frequently now compared with before you underwent chemotherapy?

From your experience, is there anything besides the topics we discussed that you feel would be important for oncology or dermatology teams to know regarding the impact of hair loss on patients?

Thank you so much for your time.
